# Preparation and Characterization of High-Strength Glass-Ceramics via Ion-Exchange Method

**DOI:** 10.3390/ma14195477

**Published:** 2021-09-22

**Authors:** Jianwei Lu, Haifeng Wang, Juanjuan Zhu, Qiuju Zheng, Linfeng Ding, Weizhong Jiang

**Affiliations:** 1 State Key Laboratory for Modification of Chemical Fibers and Polymer Materials, College of Materials Science and Engineering, Donghua University, Shanghai 201620, China; jwlu@mail.dhu.edu.cn (J.L.); whf2008@dhu.edu.cn (H.W.); zhujuanjuan@dhu.edu.cn (J.Z.); 2 Engineering Research Center of Advanced Glass Manufacturing Technology, Ministry of Education, Donghua University, Shanghai 201620, China; 3 School of Materials Science and Engineering, Qilu University of Technology (Shandong Academy of Sciences), Jinan 250353, China; qlzhengqj@163.com

**Keywords:** glass-ceramics, ion-exchange, mechanical properties, structure

## Abstract

Lithium aluminosilicate glass-ceramics (LAS GCs) are ideal shell materials for mobile phones; however, the mechanical properties of LAS GCs are comparatively lower than that of other shell materials. In this work, the impact of TiO_2_/(TiO_2_ + ZrO_2_) ratio on properties of LAS GCs was studied and the ion-exchange methods were applied to improve the mechanical properties of LAS GCs. The results show that LAS GCs with TiO_2_/(TiO_2_ + ZrO_2_) = 1/2 exhibit the best flexural strength (109 MPa) and Vickers hardness (525 Kg/mm^2^). The as-prepared glass was nucleated at 560 °C for 1 h and crystallized at 720 °C for 0.5 h. The main crystalline phases of LAS GCs are *β*-quartz solid solution, *β*-spodumene solid solution, and Li_2_SiO_3_. Moreover, the flexural strength and Vickers hardness of LAS GCs with TiO_2_/(TiO_2_ + ZrO_2_) = 1/2 further increased to 356 MPa and 838 Kg/mm^2^ after an ion-exchange at 420 °C for 6 h in pure KNO_3_ molten salt. The LAS GCs with enhanced mechanical strength have the potential to be applied as mobile phone back panels.

## 1. Introduction

Glass-ceramics (GCs) are inorganic materials with dense structure composed of microcrystalline phase and residual glass [1,2]. They combine the excellent properties of glass and ceramics. The desired properties of GCs can be obtained by adjusting the chemical composition, distribution, and proportion of crystals and glass phases [3,4]. With the development of 5G communication and wireless charging technology, the requirements of terminal equipment for shell materials are stringent [5]. Due to the drawbacks of metal in shielding signals, GCs could be a better choice as shell materials for consumer electronic devices [6].

During the preparation of GCs, the selection of nucleating agents (such as TiO_2_, ZrO_2_, or both of them) and the control of heat treatment process may change the type and content of the main crystal phase, thus affecting the thermal expansion and mechanical properties of GCs [7,8,9]. With proper nucleating agents and heat treatment, the performance of GCs can be designed by controlled crystallization.

Li_2_O-Al_2_O_3_-SiO_2_ (LAS) GCs have low coefficient of thermal expansions (CTE) and high mechanical properties due to the crystalline phases formed during thermal treatment [10,11]. The existence of crystal and glass–crystal interface makes the crack propagation pathway in GCs different from their parent glass [12,13,14]. Deng et al. [15] studied the influence of different microstructures on the crack propagation of GCs through molecular dynamics simulation, and they found that the crack propagation mode was related to the size, aspect ratio, and alignment angle of crystalline phases. Serbena et al. [16] prepared GCs with different crystallinity by controlling the heat treatment processes. With the increase in crystallinity from 0 to 100%, the mechanical properties of samples are constantly improved. They believe that the main toughening mechanisms are crack deflection and bowing and bridging caused by thermal expansion mismatch between the Li_2_O-2SiO_2_ crystal phase and residual glass. Li et al. [17] studied the effect of crystal size on the mechanical strength of lithium disilicate GCs. They concluded that the “interlocking effect’’ caused by larger-sized crystals and the “micro residual stress effect” correlated with the CTE mismatch between glass matrix and crystals simultaneously determine the flexural strength of GCs.

However, the mechanical properties of GCs still show room for improvement compared with some high-strength materials. Ion-exchange is one of the most effective methods to strengthen LAS GCs. The smaller ions on the LAS GCs surface are replaced by the larger ions in the molten salt, and then a compressive stress layer is formed on the LAS GCs surface, which greatly improves its mechanical strength [18,19]. For example, K. Laczka et al. [20] prepared the GCs with lithium disilicate and lithium aluminosilicate as the main crystal phases through proper heat treatment and the flexural strength reached 300–450 MPa. After an ion-exchange in KNO_3_ salt bath at 400–420 °C for 9 or 16 h, a surface compressive stress layer of about 15–20 μm was formed due to the exchange of potassium and sodium ions, which improved almost 100% of the mechanical resistance.

Based on the literature [21,22,23,24], the composition of glass, the consist of molten salt, and the temperature and duration of ion-exchange will influence the ion-exchange process. Thus, it is necessary to design the best ion-exchange temperature and duration according to the specific composition of LAS GCs to prepare GCs with excellent mechanical properties.

In this paper, the effects of TiO_2_/(TiO_2_ + ZrO_2_) ratio on the structure and properties of LAS GCs were investigated, and the mechanical strength of LAS GCs was further improved by the ion-exchange method. We developed a new glass-ceramic for shell materials of portable electronic devices.

## 2. Experiment Procedures

### 2.1. Sample Preparation

The following reagent-grade raw materials were used to make glasses (Sinopharm Chemical Reagent Co. Ltd., Shanghai, China): Li_2_CO_3_, Al_2_O_3_, SiO_2_, MgCO_3_, Na_2_CO_3_, ZnO, TiO_2_, ZrO_2_, NaNO_3_, KNO_3_. The parent glass compositions with different TiO_2_/(TiO_2_ + ZrO_2_) ratios are shown in Table 1. Firstly, the raw materials were melted in a quartz crucible at 1450 °C for 4 h in a furnace with MoSi_2_ heating elements (SLQ 1700, Shanghai, China). The melt was cast into a preheated iron mold to obtain a glassy block. Finally, the formed glass was annealed at 450 °C for 2 h.

**Figure 1:**
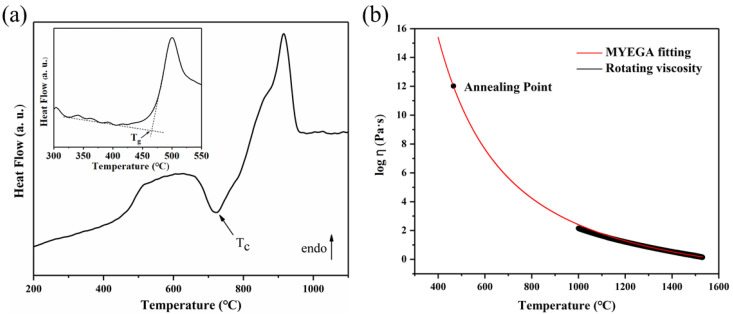
(**a**) The first DSC upscan curve of the T2 parent glass sample at a heating rate of 20 K/min. The insert figure is the second upscan curve followed by a cooling rate of 10 K/min (to determine *T*_g_); (**b**) viscosity of T2 parent glass sample measured by the rotation method. The viscosity data were fitted by the MYEGA equation.

The LAS GCs were prepared based on the measurements of T2 sample by differential scanning calorimetry (DSC, STA 449F3, Netzsch, Selb, Germany) and rotary viscometry (RSV 1600, Orton, Westerville, OH, USA). As shown in Figure 1, the viscosity–temperature curve was fitted by the MYEGA equation [25] and the glass transition temperature (*T*_g_) was obtained from the DSC method [26]. The viscosity–temperature curve provides references for the future production process. The T0-T4 specimens were nucleated at 560 °C (~100 °C higher than *T*_g_) for 1h and crystallized (around crystallization peak *T*_c_) at 720 °C for 0.5 h to obtain LAS GCs. The prepared T2 LAS GCs were further immersed in a molten KNO_3_ salt bath at 400–440 °C for different durations (2, 4, 6, 8, and 10 h).

### 2.2. Characterization

The crystalline phases of samples were identified by X-ray diffraction (XRD, D/max-2500PC, Rigaku, Tokyo, Japan). The amount of the crystalline phases (crystallized volume fraction) was determined according to the procedure used by Daguano et al. [27]. The Crystallinity Index, CI%, was calculated from the following equation:

(1) $$CI\%=\left(\frac{A_{c}}{A_{T}}\right)\times 100\%$$

where *A_c_* is the crystalline area, *A_T_* ($A_{T}=amorphous+crystalline$) is the total area.

The structure of the glass was characterized with a Fourier transform infrared spectrometer (FT-IR, Nicolet 8700, Madison, WI, USA). The CTE of LAS GCs was measured with a dilatometer (DIL 402C, Netzsch, Selb, Germany). The flexural strength of the samples was tested using a universal testing machine (WDW-20KN, Changchun, China) with a cross-head speed of 0.5 mm/min. Vickers hardness (*HV*) of the samples was tested using a microhardness tester (MHV-2000S, Shanghai, China) with a load of 0.098 N for a dwell time of 12 s. The concentration distributions of K^+^ and Na^+^ ions on the surface of LAS GCs after ion-exchange was evaluated by EDS (X-Max 50, Oxford Instruments, Oxford, UK).

## 3. Results and Discussion

### 3.1. Effect of TiO_2_/(TiO_2_ + ZrO_2_) Ratio on Properties of LAS GCs

It can be seen in Table 1 that the flexural strength of LAS GCs first increases from 86 MPa to 109 MPa with the increase in TiO_2_/(TiO_2_ + ZrO_2_) ratio, and then decreases to 81 MPa when the content of TiO_2_ reached 4 wt%. The *HV* of LAS GCs shows a similar trend to the flexural strength. Moreover, the samples change from transparent to opaque with the increase in TiO_2_/(TiO_2_ + ZrO_2_) ratio.

**Figure 2:**
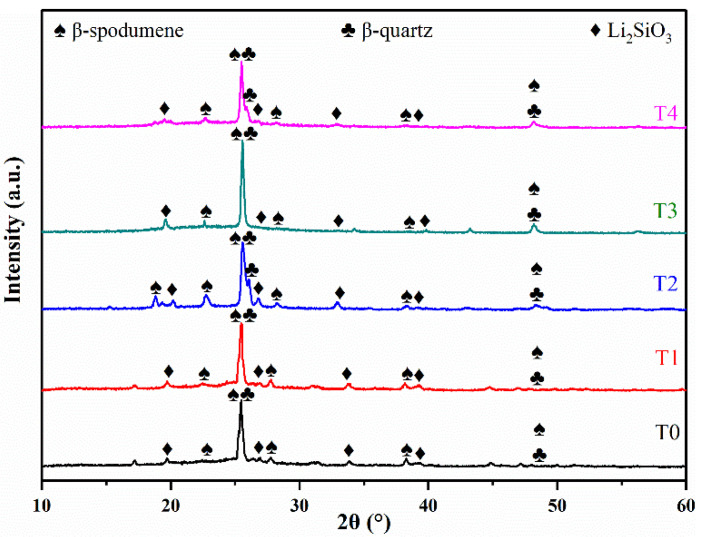
XRD patterns of T0-T4 LAS GCs.

The main reasons for the great mechanical properties of LAS GCs prepared in this work are due to the crystal species and the microstructure of GCs. The XRD results in Figure 2 show that the main crystalline phases of T0-T4 LAS GCs are *β*-quartz solid solution (LiAlSi_2_O_6_, PDF 74–1095), *β*-spodumene (LiAlSi_3_O_8_, PDF 35–794) solid solution, and lithium metasilicate (Li_2_SiO_3_, PDF 72–1140). The existence of *β*-quartz and *β*-spodumene solid solutions leads to the low CTEs of LAS GCs samples compared with conventional glasses and ceramics (e.g., the CTE of T2 sample is 3.3 ×10^−6^ °C^−1^). This low CTE is also crucial in electronic applications, especially when the heat dissipation is significant (e.g., wireless charging).

The peak intensities of T0-T4 samples are different from each other, even though the peak positions are similar. As shown in Table 1, the CI% of these samples ranges from 45% to 64%, and reaches its maximum at the TiO_2_/(TiO_2_ + ZrO_2_) ratio equal to 1/2. According to Bhattacharyya et al. [28], the ZrTiO_4_ crystal nucleus may be formed in the LAS GCs. The development of the ZrTiO_4_ crystals will enhance the heterogeneous nucleation and thus promote the crystallinity of the GC which, in turn, improved both the flexural strength and Vickers hardness [28,29].

**Figure 3:**
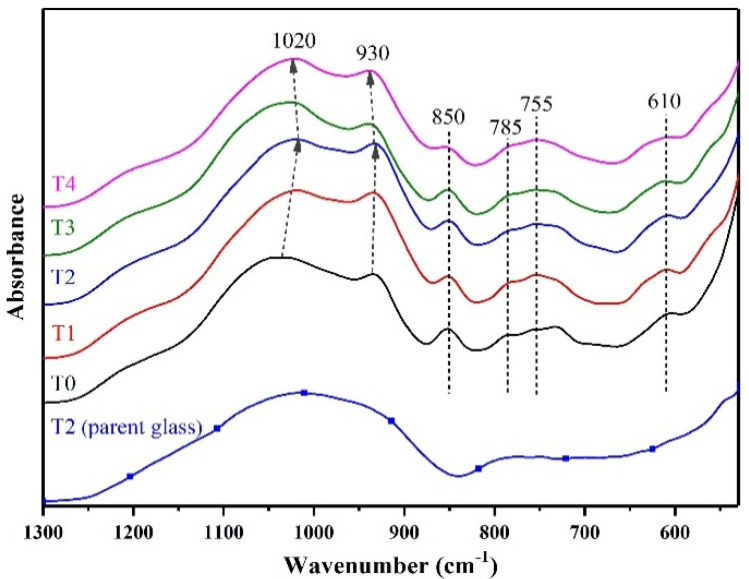
FT-IR of parent glass and T0-T4 LAS GCs.

To explore the microstructure, the FT-IR results of T0-T4 GCs are shown in Figure 3. The absorption peaks at 1020 cm^−1^/930 cm^−1^, 850 cm^−1^, and 610 cm^−1^ after crystallization can be attributed to the asymmetric stretching vibrations of Si-O-Si (Al), the symmetric stretching vibrations of Si-O-Si, and the bending vibration of Si-O-Al, respectively [30,31,32]. As can be seen in Figure 3, the broad peaks at approximately 1020 cm^−1^ and 930 cm^−1^ shift toward the lowest wavenumbers when the TiO_2_/(TiO_2_ + ZrO_2_) ratio reaches 1/2. The asymmetric stretching vibrations of Si-O-Si (Al) shifts to the lower wavenumbers when the glass network structure becomes stronger [33,34]; thus, this observation is consistent with the overall mechanical properties trend of T2 LAS GCs. Moreover, examining the XRD analysis, we find that both crystalline phases and glass network structure play important roles in the mechanical properties’ evolution in LAS GCs.

### 3.2. Ion-Exchange Strengthening Process

**Figure 4:**
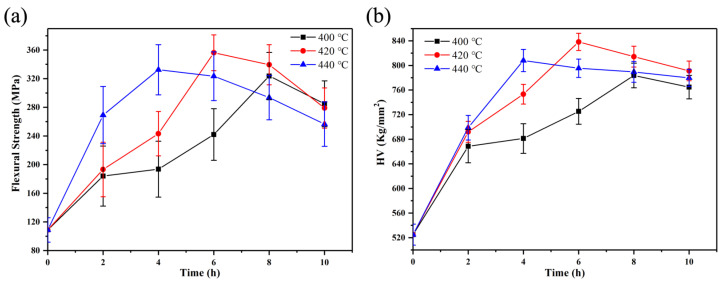
(**a**) Flexural strength and (**b**) Vickers hardness of T2 LAS GCs after ion-exchange at different temperatures for 2 h.

Figure 4 shows the flexural strength and Vickers hardness of the crystallized T2 sample (best mechanical properties) after ion-exchange at different temperatures and times. Obviously, ion-exchange improved the mechanical properties of LAS GCs. With the increase in ion-exchange temperature, it takes a shorter time to reach the highest mechanical properties. The mechanical properties of the T2 sample reach the best values after being ion-exchanged at 420 °C for 6 h, with the flexural strength of 356 MPa and Vickers hardness equaling 838 Kg/mm^2^.

**Figure 5:**
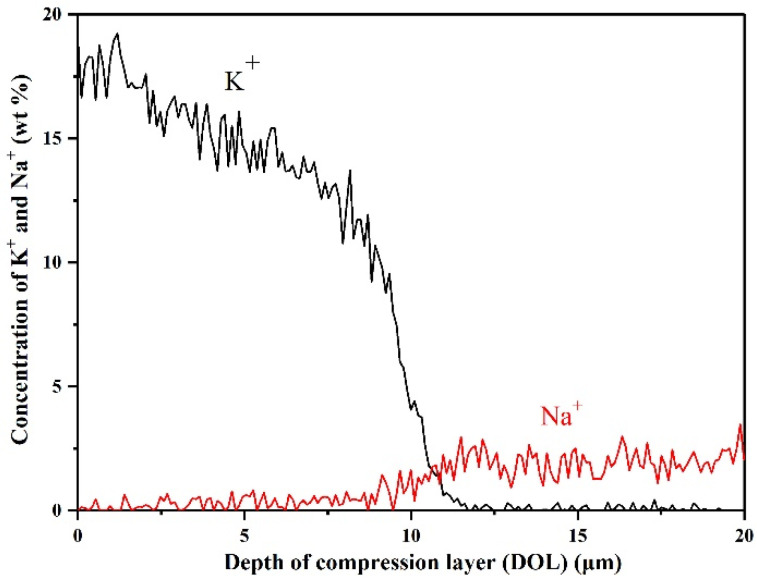
K^+^ and Na^+^ concentration distribution in T2 LAS GCs after ion-exchange at 420 °C for 2 h.

These further enhanced mechanical properties are mainly attributed to the formation of the compressive stress layer on the glass surface. Figure 5 shows the K^+^ and Na^+^ concentration distribution in T2 LAS GCs after ion-exchange at 420 °C for 2 h. The results show that the concentration of K^+^ ions gradually decreases from 16.5 wt% (on the surface) to 0 wt% (deeper than 11.7 μm inside glass body) after the ion-exchange process. Meanwhile, the concentration of Na^+^ ions increases from 0 wt% (on the surface) to 2.7 wt% (deeper than 11.7 μm inside glass body). Based on the glass compositions, it can be inferred that Na^+^, K^+^ and Li^+^ ions were all involved in the ion-exchange process because of charge balancing before and after the exchange. The smaller Li^+^ ions and Na^+^ ions on the GCs surface were replaced by the larger K^+^ ions from the molten salt, and a compressive stress layer was formed on the GCs surface, which prevents the surface crack from spreading inside to glass body under high stress [18,35]. During the ion-exchange, the exchange of Na^+^ with K^+^ ions dominate the formation of the compressive stress layer and the enhancement of mechanical properties [20].

**Figure 6:**
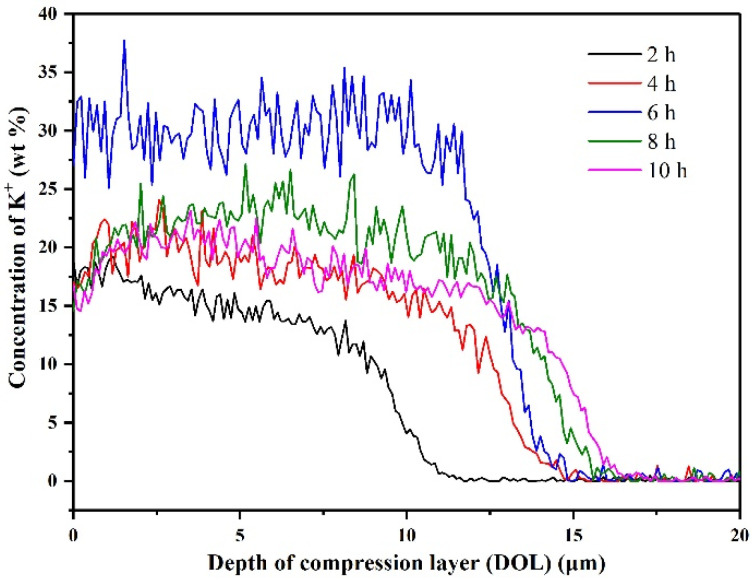
K^+^ concentration distribution in T2 LAS GCs at 420 °C for different ion-exchange times.

Moreover, the K^+^ ions diffusion profiles of LAS GCs at 420 °C for different ion-exchange times (2, 4, 6, 8 and 10 h) are shown in Figure 6. The concentration of K^+^ ions on the surface of T2 sample is very high (above 17 wt%), while it gradually decreases to 0 wt% and tends to be stable inside the glass sample. The depth of compression layer (DOL) for five different LAS GCs samples is from ~10 μm to ~17 μm. As can be seen in Figure 4, the mechanical properties of the glass are positively correlated with the concentration distribution at the first 6 h of ion-exchange. However, the “stuffing effect” [19,36] and stress relaxation of the glass structure happened afterward [37]; consequently, the mechanical properties reached the optimal at ion-exchange for 6 h at 420 °C. When the ion-exchange duration exceeded 6 h, the stress relaxation effect is dominant in the LAS GCs sample [37], and the mechanical strength of GCs starts to decrease.

## 4. Conclusions

In this study, ion-exchanged LAS GCs with high mechanical performance were prepared. The results indicate that LAS GCs with 2 wt% TiO_2_ and 2 wt% ZrO_2_, nucleated at 560 °C for 1 h, and crystallized at 720 °C for 0.5 h, exhibit the best flexural strength (109 MPa) and Vickers hardness (525 Kg/mm^2^). The main crystal phases of the prepared LAS GCs are *β*-quartz solid solution, *β*-spodumene solid solution, and lithium metasilicate. Eventually, the flexural strength and Vickers hardness further increased almost 3 times and 1.6 times after an ion-exchange at 420 °C for 6 h.

## Figures and Tables

**Table 1:** Compositions (in wt%) and properties of the LAS GCs samples.

Sample	SiO_2_	Li_2_O	Al_2_O_3_	Na_2_O	TiO_2_	ZrO_2_	Others	*HV* (Kg/mm^−2^)	Flexural Strength (MPa)	CI%	Transparency
T0	70	12.4	7.4	2.8	0	4	3.4	520 ± 21	86 ± 15	47	Transparent
T1	70	12.4	7.4	2.8	1	3	3.4	528 ± 18	101 ± 11	54	Translucent
T2	70	12.4	7.4	2.8	2	2	3.4	525 ± 17	109 ± 17	64	Opaque
T3	70	12.4	7.4	2.8	3	1	3.4	503 ± 18	96 ± 16	45	Opaque
T4	70	12.4	7.4	2.8	4	0	3.4	490 ± 25	81 ± 14	51	Opaque

## Data Availability

The data presented in this study are available on request from the corresponding author.
